# Investigating Markers of Rapport in Autistic and Nonautistic Interactions

**DOI:** 10.1089/aut.2021.0017

**Published:** 2022-03-09

**Authors:** Olivia M. Rifai, Sue Fletcher-Watson, Lorena Jiménez-Sánchez, Catherine J. Crompton

**Affiliations:** ^1^ Translational Neuroscience PhD Programme, Centre for Clinical Brain Sciences, The University of Edinburgh, Edinburgh, United Kingdom.; ^2^ Salvesen Mindroom Research Centre, Centre for Clinical Brain Sciences, The University of Edinburgh, Edinburgh, United Kingdom.; ^3^ Patrick Wild Centre, Division of Psychiatry, Centre for Clinical Brain Sciences, The University of Edinburgh, Edinburgh, United Kingdom.

**Keywords:** autism, double empathy, video coding, naturalistic communication, backchanneling, mutual gaze

## Abstract

**Background:**

Autism is considered to entail a social impairment whereby autistic people experience difficulty interpreting others' mental states. However, recent research has shown that nonautistic people also have difficulty understanding the mental states of autistic people. This mismatch of understanding may explain lower rapport in interactions between autistic and nonautistic people. As mental states can be expressed externally through socially normed signals, it is important to investigate the role of such signals in autistic, nonautistic, and mixed interactions. This study explores variability in two social signals between autistic, nonautistic, and mixed interactions, and how their use may affect rapport within interactions.

**Methods:**

Videos from a previous study of autistic, nonautistic, and mixed pair interactions in a diffusion chain context in which participants were aware of others' diagnostic status were video coded for mutual gaze and backchanneling as candidate indicators of interactional rapport.

**Results:**

Although use of mutual gaze and backchanneling was lower in mixed pairs than in nonautistic pairs, corresponding to lower ratings of interactional rapport, less backchanneling in autistic pairs of both nonverbal and verbal subtypes corresponded to higher ratings of rapport.

**Conclusions:**

We observed differences in the use of candidate rapport markers between autistic, mixed, and nonautistic interactions, which did not map onto patterns of rapport scores, suggesting differences in reliance on these cues between autistic and nonautistic people. These results suggest that visible markers of rapport may vary by neurotype or pairing and give clues to inform future investigations of autistic interaction.

## Community brief

**Why is this an important issue?:**

When someone is autistic, society generally assumes they have difficulty interacting with others. Social difficulties between autistic and nonautistic people are thought to be due to the autistic person not being able to interact using nonautistic social behaviors. This belief can lead to many autistic people feeling alienated. However, recent research supports what autistic people have been saying for a long time: that autistic people are capable of having successful and rewarding interactions with other autistic people. This suggests that social difficulties between autistic and nonautistic people may be due to a mismatch in social norms leading to difficulty for both people, not just the autistic person.

**What was the purpose of this study?:**

In this study, we wanted to investigate whether certain social signals are used differently between autistic and nonautistic people. We also questioned whether using these signals helped or hurt the interaction depending on who was involved.

**What did the researchers do?:**

We recorded people passing a story down a chain of people, like the game telephone, to see how they interacted with each other. Afterward, people wrote down scores for how much they enjoyed their interaction. We focused on two parts of the interaction: how much people were looking at each other and when they made short verbal responses such as “mhm” to show they were listening (backchanneling). We watched the recordings back and analyzed how long or how many times these actions were occurring.

**What were the results of the study?:**

We found that when one autistic and one nonautistic person were interacting, they looked at each other and backchanneled less than two nonautistic people. This seemed to be linked with a less enjoyable interaction for them. However, backchanneling seemed to matter less in interactions between two autistic people. They backchanneled less while still having enjoyable interactions.

**What do these findings add to what was already known?:**

Research has previously suggested that different social norms exist between autistic and nonautistic people. This study shows specific examples of this and how they may affect the interaction in a natural setting.

**What are potential weaknesses in the study?:**

This study has some weaknesses. For example, we measured when people looked at each other's faces rather than eye contact specifically, which can only be done with an eye tracker. Also, the people in the study knew whether the person they were talking to was autistic. This can be similar to normal life, as people do sometimes know this, although we would also be interested to see what the effect of not knowing would be.

**How will these findings help autistic adults now or in the future?:**

This line of research has important implications for how autistic people can be supported in society. Not only will understanding of social differences between autistic and nonautistic people help the way autistic people are perceived, but it will also help nonautistic people better understand and support the autistic people in their lives.

## Introduction

Autism is considered an inherently social impairment.^1^ As such, a large portion of autism research focuses on mechanisms by which autistic people experience social difficulty.^2^ In particular, autistic people's perceived inability to interpret others' mental states has led to the pervasive claim that autistic people lack a Theory of Mind.^3,4^ However, empirical failures of Theory of Mind investigations have recently been highlighted,^5^ and there is ongoing controversy as to how Theory of Mind is defined.^6^ Moreover, research has demonstrated that nonautistic people also have difficulty interpreting mental states of autistic people,^7,8^ despite believing that they are socially competent and helpful when interacting with autistic people.^9,10^ Thus, Theory of Mind, and the assumption that autistic people are socially impaired, fails to acknowledge that the success of a social interaction depends on two people.

In contrast, the Double Empathy Problem reframes social difficulties between autistic and nonautistic people at the level of the pair, rather than an individual,^11^ and suggests that difficulties arise due to a mismatch between autistic and nonautistic social expectations. This is supported by recent research that found evidence of successful interactions between autistic people, as indexed by information transfer and self-rated rapport.^12^ Autistic people qualitatively report that interacting with other autistic people is more comfortable and less tiring than interacting with nonautistic people,^13,14^ and experience higher rapport when interacting with other autistic people compared with nonautistic people.^15^

Together, these data raise new questions about *how* social rapport is achieved in autistic interactions. There are indications that autistic people experience intersubjectivity, or mutual understanding, through shared social norms.^16,17^ It is suggested that dialectical misattunement, or a mismatch of social norms leading to a divergence in communication style over time, may be a mechanism behind the Double Empathy Problem, and further, that similarities in communication style between autistic people may contribute to intersubjectivity.^18,19^ However, more empirical investigations are needed to inform the specific question of whether the same social cues facilitate autistic and nonautistic interactions. In this study, we analyze video data from a previous study^12^ to investigate the existence of observable social cues that act as markers of rapport within these interactions, and whether they differ between autistic, nonautistic, and mixed pairs. This previous study involved task-based interactions between autistic, nonautistic, and mixed autistic/nonautistic pairs, in which participants were aware of each other's diagnostic status.

Rapport has been previously conceptualized as a quality of the interaction or relationship between two people, based on elements such as attentiveness, positivity, and coordination.^15,20,21^ These factors have been used to measure rapport in previous studies^21^ and are thought to be important components of successful interaction.^22^ Although most psychological constructs examine individual performance, rapport relates to the interaction and coordination between two people.^23^ Rapport is built and enhanced through both nonverbal (e.g., mutual gaze, facial expressions, and postural mirroring) and verbal behaviors (e.g., backchanneling and tone of voice),^20,24^ with lower rapport associated with an absence of these behaviours.^25–27^ This considered, two candidate markers of rapport were chosen for this study: mutual gaze and backchanneling, based on their possible contributions to elements of rapport. Eye contact is an important component of “successful” nonautistic interaction that can help one perceive information from others, such as the desire to communicate and prompts for turn-taking, as well as establish joint attention.^28,29^ For this study, we have employed a more general surrogate measure of eye contact that could be measured from video data, that is, the extent to which participants are looking at each other's faces, herein referred to as “mutual gaze.” Backchanneling is a response by the listener that conveys attentiveness and impacts how well the speaker feels they are being understood. Backchanneling manifests through nonverbal elements such as nods, or verbal elements such as “mmhmm.”^30,31^ These rapport markers were examined in video data of paired interactions and evaluated against self-rated rapport scores to determine whether they can be considered viable objective markers of rapport for either nonautistic, autistic, or mixed pairs.

## Methods

### Participants

We enrolled 72 adults in the original study^12^: 24 in each of the autistic, nonautistic, and mixed (12 autistic and 12 nonautistic) groups. Participants took part in a diffusion chain: a paradigm in which novel information or skills are passed from one person to the next, over a series of paired interactions.^12^ For the following analyses, we removed the final pair in each of the diffusion chains before video coding, because due to the nature of the task and the degradation of information between pairs, the last pair in each chain had a very short and variable interaction (mean 50 seconds; range 35–71 seconds) that was significantly less task-oriented, leading this interaction to be too unreliable for inclusion in our comparisons. This removed 9 participants' data entirely, because they occupied the final place in the chain, leaving a total of 63 participants (21 in each group) (Table 1).

**Table 1.** Descriptive Statistics [Mean (Standard Deviation)] on Demographics by Group, Using Kruskal–Wallis Chi Square, Analysis of Variance, and Fisher's Exact Test Comparisons

	Nonautistic (*n* = 21)	Autistic (*n* = 21)	Mixed (*n* = 21)	Comparisons
Age	34.62 (12.06)	35.57 (11.03)	33.10 (9.04)	χ^2^(2) = 0.55, *p* = 0.78
Gender	18 F, 3 M	17 F, 1 M, 3 NB	16 F, 5 M	Fisher's exact test *p* = 0.10
Years of education	17.76 (1.45)	17.50 (2.77)	17.26 (1.89)	χ^2^(2) = 1.02, *p* = 0.55
IQ—WASI—II	114.48 (11.54)	116.24 (15.32)	117.62 (13.83)	*F*(2,60) = 0.28, *p* = 0.76
Autism quotient	13.50 (5.92)	36.05 (6.30)	24.86 (14.09)^a^	χ^2^(2) = 28.02, *p* < 0.0001
Age of diagnosis	NA	31.22 (11.46)	29.75 (10.28)	χ^2^(2) = 0.20, *p* = 0.67

^a^ This mean includes both autistic and nonautistic participants; when subdivided the means (standard deviation) are 14.58 (6.83) for nonautistic participants and 38.56 (7.92) for autistic participants.

IQ, intelligence quotient; NB, nonbinary; WASI—II, Wechsler Abbreviated Scale of Intelligence—II.

We matched groups on age, gender, years of education, and intelligence quotient (IQ) (Table 1). All participants spoke English to a native level and did not have a diagnosis of social anxiety disorder. Participants completed the Wechsler Abbreviated Scale of Intelligence—II (WASI—II),^32^ a measure of IQ, with all participants scoring within a typical range. Nonautistic participants scored <32 on the Autism-Spectrum Quotient^33^ and autistic participants had a clinical diagnosis or scored >72 on the Ritvo Autism-Asperger Diagnostic Scale-Revised (RAADS-R)^34^ if self-diagnosed (*n* = 3).

### Procedure

We conducted the study as per the British Psychological Society's Code on Human Research Ethics after ethical review and approval by the University of Edinburgh Psychology Research Ethics Committee. Participants gave written informed consent before study participation. Participants completed a diffusion chain task that involves a series of dyadic interactions. A researcher tells Participant A a fictional story; Participant A then shares this story with Participant B. Participant B then shares this story with Participant C and so on, until Participant H. This generated seven paired interactions between participants in each chain. Only the two participants sharing the story were in the same room at any time. We informed participants before starting the interactions whether they were in an autistic, nonautistic, or mixed pair. The decision to make participants aware of the diagnostic status of their interaction partner was threefold: first, in some real-world contexts, diagnostic status may be known between individuals (e.g., peer-support groups, healthcare, and educational settings); second, to increase participant comfort level and reduce any additional stress associated with research participation and/or interacting with unfamiliar people; and third, as disclosing diagnostic status could arise in conversation between participants during the tasks, disclosing diagnostic status in advance ensured that all participants had the same information. Participants did not know each other before the study began and stayed in separate rooms throughout the study when not engaged with the diffusion chain tasks. Immediately afterward, participants rated their experience of rapport with their partner on a Likert-type five-dimensional rapport scale^15^ designed based on core rapport domains described previously,^20,21,35^ where ease, enjoyment, success, friendliness, and awkwardness (reverse-scored) were rated between 0 and 100 (Supplementary Data).

### Defining variables

#### Paired-mean rapport

The five dimensions of the rapport scale had a high Cronbach's alpha (0.71), so we summed them to create a single scale of interactional rapport. We report self-rated rapport at the pair level, that is, averaged self-rated rapport of both participants in each pair, herein referred to as “paired-mean rapport,” to analyze the relationship between rapport and other pair-level data in the study. We include a new rapport analysis that excludes the final pair in each chain, compared with that which has been published previously.^12^ Intrapair rapport correlations can be found in Supplementary Figure S1.

#### Mutual gaze and backchanneling

A researcher, blinded to diffusion chain type, performed video coding using the video annotation software ELAN (version 4.9.2)^36^ for mutual gaze and backchanneling. For the coding scheme, we defined mutual gaze as the time during which participants are looking at each other and measured this as a percentage of the interaction duration to control for varying interaction times. As we did not perform eye-tracking in the original study, this variable relied on the coder's subjective perception of eye-gaze direction, coding for looking at the face of their partner. We defined backchanneling as a response of the listener that conveys attention or understanding to the speaker, delineated by responses to new verbal information and measured as a rate, that is, frequency of backchanneling instances ÷ interaction duration. We chose to measure frequency of backchanneling, rather than elements such as backchanneling tone, as frequency would be the most objective, and therefore robust, measure for an exploratory analysis. We coded both verbal and nonverbal backchannels. The details of the full coding scheme are available in Supplementary Data. To calculate inter-rater reliability, a second independent researcher coded one-third of the data set. Mutual gaze duration between coders had an intraclass correlation of 0.994, calculated using “irr” for R,^37,38^ indicating excellent agreement.^39^ For backchanneling counts, the two coders had 98.8% agreement (Cohen's *k* = 0.94) for nonverbal and 98.5% agreement (Cohen's *k* = 0.85) for verbal backchanneling, again demonstrating high reliability^40^ (Supplementary Data).

### Statistical analysis

We conducted statistical analyses to determine differences between groups, with nonautistic as the reference group, using GraphPad Prism (version 8.0.0).^41^ An *a priori* power analysis run in “pwr” for R^38,42^ indicated that our sample size gave us 90% power to detect a medium effect (0.5) at the standard alpha error probability (0.05) for pair-level data, that is, paired-mean rapport, mutual gaze duration, and backchanneling rate within pairs (*n* = 54). We removed outliers more than two standard deviations (SDs) away from the mean of each group (for paired-mean rapport, one pair from each of the nonautistic and autistic groups; for mutual gaze, one pair from each group; for backchanneling, one pair from each of the mixed and autistic groups), and we checked data for normality and homogeneity using Shapiro–Wilk's and Bartlett's tests, respectively.

We analyzed normal and homogeneous data with ordinary one-way analysis of variance (ANOVA) and Dunnett's tests, normal and nonhomogeneous data with Welch's one-way ANOVA and Dunnett's T3 tests, and non-normal data with Kruskal–Wallis nonparametric ANOVA and Dunn's tests. We conducted comparisons between mixed and autistic pairs using tests that compared all group means. We reported adjusted *p*-values from the aforementioned multiple comparisons tests (Dunnett's, Tukey's, Dunnett's T3, or Dunn's) and considered *p*-values <0.05 to be significant. We computed effect sizes (Cohen's *d*) using group mean differences and a pooled SD calculated as *d* = (M_2_ − M_1_) ⁄ SD_pooled_ where SD_pooled_ = √((SD_1_^2^ + SD_2_^2^) ⁄ 2). For Supplementary correlation analyses in Supplementary Data, we correlated mutual gaze duration and backchanneling rate with paired-mean rapport and reported Pearson's and Spearman's coefficients for normal and non-normal data, respectively. We refer to correlations as moderate (±0.4–0.6), weak (±0.1–0.3), or no correlation (±0.0–0.1).^41^

## Results

Paired-mean rapport scores significantly differed between the three groups (ordinary one-way ANOVA, *p* < 0.001): Mixed pairs had significantly lower rapport than nonautistic pairs (Dunnett's test, *p* < 0.001) and autistic pairs (Tukey's test, *p* = 0.007), whereas autistic pairs did not significantly differ from nonautistic pairs (Dunnett's test, *p* = 0.1030) (Table 2 and Supplementary Fig. S1).

**Table 2.** Paired-Mean Rapport by Pair Type After Removal of Outliers

	Mean	Standard deviation	*n*
Nonautistic pairs	405.52	54.68	17
Mixed pairs	333.38, NA: 374.00, A: 289.94	42.07, NA: 40.67, A: 64.75	18, NA: 9, A: 9
Autistic pairs	375.41	52.83	17

A: rating the autistic participant provided within mixed pairs; NA: rating the nonautistic participant provided within mixed pairs.

**FIG. 1.**
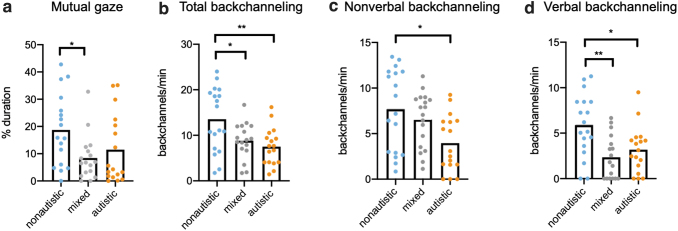
Mutual gaze and backchanneling levels in nonautistic, mixed, and autistic pairs. **(a–d)** Mean and individual values shown by pair type for **(a)** mutual gaze duration %, **(b)** total backchanneling rate, **(c)** nonverbal backchanneling rate, and **(d)** verbal backchanneling rate (*p < 0.05, **p < 0.01).

Group results for mutual gaze duration and backchanneling rates are listed in Table 3. For mutual gaze, Kruskal–Wallis ANOVA revealed a significant main effect of pair type (*p* = 0.0434); the duration was significantly shorter in mixed pairs (Dunn's test, *p* = 0.0482, *d* = 0.94), but not in autistic pairs (Dunn's test, *p* = 0.0767, *d* = 0.56) compared with nonautistic pairs (Fig. 1a); autistic and mixed pairs did not significantly differ (Dunn's test, *p* = 0.9999, *d* = 0.29). There was a moderate positive correlation between mutual gaze duration and paired-mean rapport (Pearson's *r* = 0.4460, *p* = 0.042) for nonautistic pairs, whereas correlations were nonsignificant for mixed (Spearman's *r* = 0.4093, *p* = 0.052) and autistic pairs (Spearman's *r* = 0.2059, *p* = 0.221) (Supplementary Fig. S2a).

**Table 3.** Mutual Gaze Duration and Backchanneling Rates by Pair Type After Removal of Outliers

	Nonautistic pairs	Mixed pairs	Autistic pairs
Mutual gaze duration^a^	18.71 ± 13.16 (*n* = 17)	8.44 ± 8.25 (*n* = 17)	11.49 ± 12.66 (*n* = 17)
Total backchanneling^b^	13.55 ± 6.99 (*n* = 18)	8.80 ± 3.94 (*n* = 17), NA: 8.90 ± 3.81 (*n* = 9), A: 8.69 ± 4.34 (*n* = 8)	7.51 ± 4.09 (*n* = 17)
Nonverbal backchanneling^b^	7.67 ± 4.48 (*n* = 18)	6.53 ± 3.00 (*n* = 18), NA: 6.60 ± 3.31 (*n* = 9), A: 6.47 ± 2.85 (*n* = 9)	3.96 ± 6.09 (*n* = 17)
Verbal backchanneling^b^	5.88 ± 3.45 (*n* = 18)	2.35 ± 2.39 (*n* = 17), NA: 2.30 ± 2.23 (*n* = 9), A: 2.40 ± 2.71 (*n* = 8)	3.18 ± 2.57 (*n* = 17)

A: autistic participant data within mixed pairs; NA: nonautistic participant data within mixed pairs.

^a^ Measured as a percentage of task duration.

^b^ Measured as a rate (backchannels per minute).

For backchanneling rate, Welch's ANOVA revealed a significant main effect of pair type (*p* = 0.0145); the rate was significantly lower in mixed pairs (Dunnett's T3, *p* = 0.0377, *d* = 0.84) and autistic pairs (Dunnett's T3, *p* = 0.0079, *d* = 1.05) compared with nonautistic pairs (Fig. 1b); autistic and mixed pairs did not significantly differ (Dunnett's T3, *p* = 0.7235, *d* = 0.32). These findings were recapitulated and extended when we examined each backchanneling modality separately. There was a significant main effect of pair type on both nonverbal and verbal backchanneling rates independently (Kruskal–Wallis ANOVA, *p* = 0.0123; Kruskal–Wallis ANOVA, *p* = 0.0050). Nonverbal backchanneling was significantly reduced in autistic pairs (Dunn's test, *p* = 0.0085, *d* = 0.97), but not in mixed pairs (Dunn's test, *p* = 0.9392, *d* = 0.30), compared with nonautistic pairs, and was not significantly different between mixed and autistic pairs (Dunn's test, *p* = 0.0952, *d* = 0.85). Verbal backchanneling was significantly reduced in both autistic (Dunn's test, *p* = 0.0404, *d* = 0.89) and mixed (Dunn's test, *p* = 0.0036, *d* = 1.18) pairs compared with nonautistic pairs (Fig. 1c, d); autistic and mixed pairs did not significantly differ (Dunn's test, *p* = 0.9999, *d* = 0.35). Correlations between backchanneling rate and paired-mean rapport were nonsignificant (nonautistic pairs: Pearson's *r* = 0.1344, *p* = 0.304, mixed pairs: Pearson's *r* = 0.1246, *p* = 0.317, autistic pairs: Pearson's *r* = −0.2167, *p* = 0.210) (Supplementary Fig. S2b).

## Discussion

**FIG. 2.**
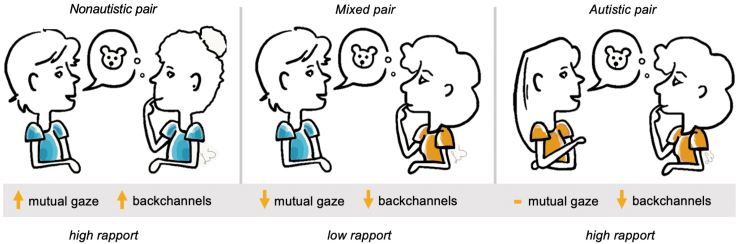
Summary of results across pair types. Mutual gaze duration % and backchanneling rate are indicated as high in nonautistic pairs with upward arrows. In mixed and autistic pairs a downward arrow indicates lower use of a particular signal compared with nonautistic pairs, whereas a dash indicates no significant difference.

This exploratory analysis sought to examine potential markers of rapport in autistic, nonautistic, and mixed interactions. Mutual gaze and backchanneling varied between pair types, with both mixed pairs and autistic pairs exhibiting a significantly lower amount of backchanneling, and mixed pairs exhibiting a lower amount of mutual gaze (Fig. 2). Although nonsignificant, autistic pairs had lower mutual gaze than nonautistic pairs; as this result was on the borderline of significance (*p* = 0.0767), it would be interesting to replicate this study in a larger sample to examine whether this would become a significant or nonsignificant result. In addition, using a more sensitive technology such as eye-tracking may provide more information about the role of eye contact in autistic interactions.

While less use of one or both of these established nonautistic signals of attentiveness was associated with lower rapport in mixed pairs, this was not the case for autistic pairs. Interestingly, in our exploratory correlation analysis (which must be interpreted with caution due to low power), we observed a significant correlation between mutual gaze duration and paired-mean rapport in nonautistic pairs, which was not maintained for mixed or autistic pairs. Our preliminary results suggest that successful autistic interactions may be less reliant on nonautistic social norms than nonautistic interactions. It is possible that a mismatch in mutual gaze or backchanneling use between autistic and nonautistic participants may contribute to the exceptionally lower rapport reported by mixed pairs, in support of the Double Empathy Problem, although further research will be required to examine alternative mechanisms.

This study has some limitations. First, it is important to consider the imprecision of our mutual gaze classification. It is possible that people may be looking at their partner's mouth and not their eyes, which cannot be confirmed without using a more precise measure such as eye-tracking, and this might be more common for autistic people.^43^ Thus, it could be expected that the use of an eye tracker might reveal a lower amount of mutual gaze in autistic pairs than reported in this study. However, the extent to which looking at mouths would affect how someone feels they are being looked at, and potentially related feelings of rapport, is unclear; if nonautistic people view this behavior as unnatural, it may negatively impact their perception of the autistic person.^44,45^ Second, participants were not blind to others' diagnostic status. As in some real-world contexts, diagnostic status may be known (e.g., speaking with a family member) and in others it may be unknown (e.g., asking a stranger a question), it will be important to replicate the study with participants unaware of their partner's diagnosis. Diagnostic disclosure has been found to improve first impressions of autistic adults,^46^ and both autistic and nonautistic raters formed less favorable impressions of autistic than nonautistic speakers when blind to diagnostic status.^47^ Thus, it may be that rapport scores would be even lower in mixed pairs and lower in autistic pairs when blind to diagnostic status; however, definitive in-group/out-group knowledge would no longer be an influence. Future research could examine the extent to which these factors counterbalance in blind and unblind contexts.

Third, due to our diffusion chain design, as each participant featured in two pairs, the data are partially dependent. This means that, for example, if a participant rates rapport highly, this will affect two dyad observations, and so an outlier could have disproportionately high effects. We attenuated this effect by excluding outliers beyond two SDs. The design also does not allow us to determine whether individual participants are changing the amount of signaling used based on the neurotype they are paired with, as participants are different between nonautistic, mixed, and autistic groups. Future studies may replicate this paradigm in a larger sample with either independent dyads, or with each participant interacting with both an autistic and nonautistic person, to gain further insight into mechanisms underlying social interaction. Fourth, men are underrepresented in the study, and all participants had an IQ within the typical range, limiting generalizability to the wider autistic population. Autistic men have been hypothesized to perform less masking, that is, altering of their natural behavior to appear more nonautistic,^48^ so it is possible that rapport scores would be affected if more men were included. In addition, although fully powered to detect effects, our sample size was not sufficient to analyze the effect of factors such as gender and age on interaction. Finally, we did not collect information on ethnicity in the original study. Importantly, autistic people of color are underrepresented in autism research and may face barriers and biases in everyday interactions^49^; thus, it is necessary that future studies collect such data to allow us to understand how rapport interacts with ethnicity in autistic and nonautistic interactions.

Despite these limitations, the results provide a promising basis for further investigation and raise novel questions. The memory-related nature of the task might have contributed a high cognitive load, or demand imposed on working memory, to which autistic people are more sensitive.^50–52^ This potential effect may reduce mutual gaze and backchanneling use more so than in casual conversation; thus, it may be interesting to explore other interaction contexts. There are also several other potential markers worth analyzing, such as facial expressions, hand gestures, interruptions, postural mirroring, word repetition, prosody, repair sequences, coherence of conversational turns, sudden movements, and general interpersonal synchrony,^10,44,45,53^ which were excluded from this study as they were either too sparse in the available footage or would require more advanced technology to be measured effectively. We hope that these questions can be addressed in future studies of a larger scale.

Research examining autistic interactions and their underlying mechanisms is increasingly challenging traditional assumptions of autistic social impairment. Our results suggest that autistic rapport may not depend on social behaviors considered to be “rapport building”; for example, autistic pairs had relatively high levels of rapport alongside lower instances of backchanneling. Such autistic social differences may be perceived as social *faux pas* by nonautistic people based on their established set of social norms, thereby othering autistic people who operate differently.^54^ If difficulties in social communication between nonautistic and autistic people can be thought of as a shared hurdle^55^ of mismatched social expectations rather than the fault of the autistic person, perhaps the effects of such othering felt by autistic people can be ameliorated over time. Furthermore, enhancing our understanding of what makes a good “autistic” interaction by identifying specific markers, or lack thereof, may help us better facilitate successful interactions between autistic and nonautistic people. Knowledge of how autistic people use social cues and how they shape their experience of an interaction should be deployed by professionals working with autistic people, especially when the role relies to a large degree on interpersonal rapport, for example, in psychiatric services. More generally, we should cultivate a culture of adaptation, through which nonautistic people are educated about autistic norms, to better support and understand the autistic people in their lives.

## Supplementary Material

Supplemental data

Supplemental data

Supplemental data
