# Supplementary material for: Investigating Markers of Rapport in Autistic and Nonautistic Interactions
Source: Autism Adulthood. 2022 Mar 9;4(1):3–11. doi: 10.1089/aut.2021.0017 (PMC8992924; doi:10.1089/aut.2021.0017)
Supplement: Supplemental data [file Supp_DataS1.docx]

**Supplemental Information**

**Five-dimensional rapport scale survey given to participants**

Today, you’ll be asked to complete some scales about how you felt during different tasks. After each task, you’ll be given a set of questions for example “How much did you enjoy interacting with this person?” Underneath each question will be a line, with 0 marked at the left side, and 100 marked on the right side, like this.

0

100

A score of 100 means that you had an easy, friendly interaction. This may have made you feel happy, perhaps it felt natural to you, or comfortable.

A score of 0 means that you had a very difficult interaction. Perhaps you had problems understanding one another while doing the task. You might have felt anxious or very confused.

You can score your answers anywhere between 0 and 100. You should mark your answer with a cross. For example, if you found interacting with someone pretty difficult and you didn’t enjoy it very much, you could complete your answer like this

“How much did you enjoy interaction with this person?”

100

0

If you got on really well with someone and really enjoyed completing the task with them, you could complete your answer something like this

“How much did you enjoy interaction with this person?”

100

0

Please note that you’ll be given two sets of questions after each task – one set about each person that you interacted with during the task.

If you have any questions about this, please ask one of the research team.

How much did you enjoy interacting with this person?

100

0

How easy did you find interacting with them?

0

100

How successful did you think the interaction with this person was?

0

100

How friendly did you find the interaction?

100

0

How awkward did you find the interaction?

0

100

**Coding scheme development**

Variables to be captured by the coding scheme were selected after researching physical and verbal conversational elements that may affect feelings of rapport between conversational partners and conducting a preliminary watch of the videos to determine whether such elements could feasibly be coded for given the available footage (unpublished method, Nordahl-Hansen & Fletcher-Watson). The scheme was established based on 5-minute conversation videos and how markers would be most effectively measured using these data. The final scheme included gaze segments, conversational turns, and backchannelling (non-verbal and verbal), and was applied to storytelling diffusion chain videos. Coding was only performed on the active storytelling sample and not extraneous footage, for participant pairs 1-6 and not 7, due to paucity of codable footage at the final pairing in the chain. Average duration of a pair interaction was ~5 minutes for conversations and ~2 minutes for storytelling.

**Video coding scheme for gaze (duration).**

|  | Included | Excluded |
| --- | --- | --- |
| When the eyes are visible | Any segments where the eyes are directed at the other participant’s face, blinks or other closing of the eyes for less than 0.5 s | Any segments where the eyes are directed somewhere other than the participant’s face, closing of the eyes for longer than 0.5 s |
| When the eyes are obscured (by dark sunglasses, hair, hands) | Any segments where the face of the participant is facing the other participant’s face | Any segments where the face of the participant is not facing the other participant’s face |

**Video coding scheme for speaking (duration).**

|  | Included | Excluded |
| --- | --- | --- |
| Storytelling task | Telling of the story starting from the first phrase that contains the story and ending with the last phrase that contains the story and an indication that the story has been finished (e.g. “And that’s the story” or a nod or hand gesture) | Backchannels (non-verbal, verbal – *see criteria for backchannels*), any comments or re-telling of the story after the initial telling is finished, the pairing at the beginning that includes the investigator, the ending where the last participant tells the story to the camera |

**Video coding scheme for backchannelling (rate).**

|  | Included | Excluded |
| --- | --- | --- |
| Non-verbal backchannels | *When the participant is the listener* - Nodding, shaking the head | Head tilts, facial expressions (e.g. smiles), hand gestures, anything that would be considered a backchannel except the participant is not the listener and therefore is not conveying attentiveness |
| Verbal backchannels | *When the participant is the listener* - Laughs (breathy, vocal), non-lexical elements (e.g. mhm), lexical elements (e.g. wow), repeated words/phrases, short comments that are not part of a conversational turn | Unsuccessful interruptions, requests for clarification, general questions, comments that are part of a conversational turn, anything that would be considered a backchannel except the participant is not the listener and therefore is not conveying attentiveness |

For the storytelling diffusion chains, ‘Gaze’, ‘Talk’ and ‘BC’ (i.e. backchannelling) types were created. The tiers for each type were created per participant as follows: gaze was counted per participant per conversational phase, i.e. speaker and listener, talk was counted per participant only when the participant is the speaker, and backchannelling was counted per participant only when the participant is the listener. For example, participant 2 would have tiers ‘P2-Gaze-listener’, ‘P2-Gaze-speaker’, ‘P2-speak’, ‘P2-nonverbal-BC’ and ‘P2-verbal-BC’. Durations of gaze and speaking were initially recorded in Segmentation Mode using two keystrokes per annotation with a keystroke delay of 1000 ms. Segments were refined in Annotation Mode using the Alt key to drag initial segment boundaries. Non-verbal and verbal backchannelling frequencies were initially recorded in Segmentation Mode using one keystroke per annotation with a fixed duration of 1000 ms and a keystroke delay of 1000 ms. Segments were refined in Annotation Mode using the Alt key fix initial segment positions (without changing the size of the segment from 1000 ms). Backchannels were marked so that the beginning of each 1000 ms segment coincided with the beginning of each backchannel.

All data were exported as tab-delimited text files with a time column for ‘Begin Time’, ‘End Time’ and ‘Duration’ with the time format including seconds and milliseconds (ss.msec). These data were opened in Notepad and copied into Microsoft Excel 2016. Much of the analysis involved measuring the overlap between tiers, which was done by creating a new tier under Create Annotations from Overlaps, (1) checking the desired tiers, (2) regardless of their annotation overlaps, (3) marking the type, and (4) value in the time format ss.msec. More details about overlap tiers and normalisation for each measurement type can be found below.

**Protocols for making overlap tiers and exporting ELAN data. (normalisation calculations)**

| Measurement | Protocol |
| --- | --- |
| Mutual and total gaze (duration %) | An overlap tier was created between gaze of both participants to measure mutual gaze. The mutual gaze, total gaze and talk tiers were exported. The sum of mutual gaze was divided by the sum of talk of both participants to calculate the percentage of the conversation time that mutual gaze occurred. The sum of total gaze per participant was divided by the sum of talk of both participants to calculate the percentage of the conversation that each participant was looking at the other participant. |
| Backchannelling (rate) | Non-verbal backchannelling, verbal backchannelling and talk tiers were exported. The number of rows in the backchannelling datasets per participant (total) or per participant per type (non-verbal versus verbal) were calculated and divided by the sum of talk of the other participant (listening time) to find the number of backchannels per minute of listening. |

**Calculating inter-rater reliability**

Inter-rater reliability was calculated using one-third of the dataset (one “set” of autistic, non-autistic and mixed participants). Data from ELAN were exported into Excel and speaking segments were compared between coders to establish a “master” speaking segment defined by the overlap of speaking segments between coders, within which the following calculations were performed. As mutual gaze duration is a continuous variable, we determined the intraclass correlation between mutual gaze duration coded by two independent researchers using a two-way random effects model in the package “irr” for R. As backchannelling counts are categorical data (backchannel or not backchannel), we calculated % agreement and Cohen’s *k* using an online calculator at <https://idostatistics.com/cohen-kappa-free-calculator/>. Backchannel agreement was measured by dividing speaking segments into units of 1 second and determining which backchannels were coded by both speakers (with a 1.5 second grace period) and which were coded by only one speaker; the remaining seconds were counted as “not backchannel”. Backchannelling was divided into non-verbal and verbal backchannelling for this analysis.
