# Supplementary material for: Investigating Markers of Rapport in Autistic and Nonautistic Interactions
Source: Autism Adulthood. 2022 Mar 9;4(1):3–11. doi: 10.1089/aut.2021.0017 (PMC8992924; doi:10.1089/aut.2021.0017)
Supplement: Supplemental data [file Supp_FigS1.docx]

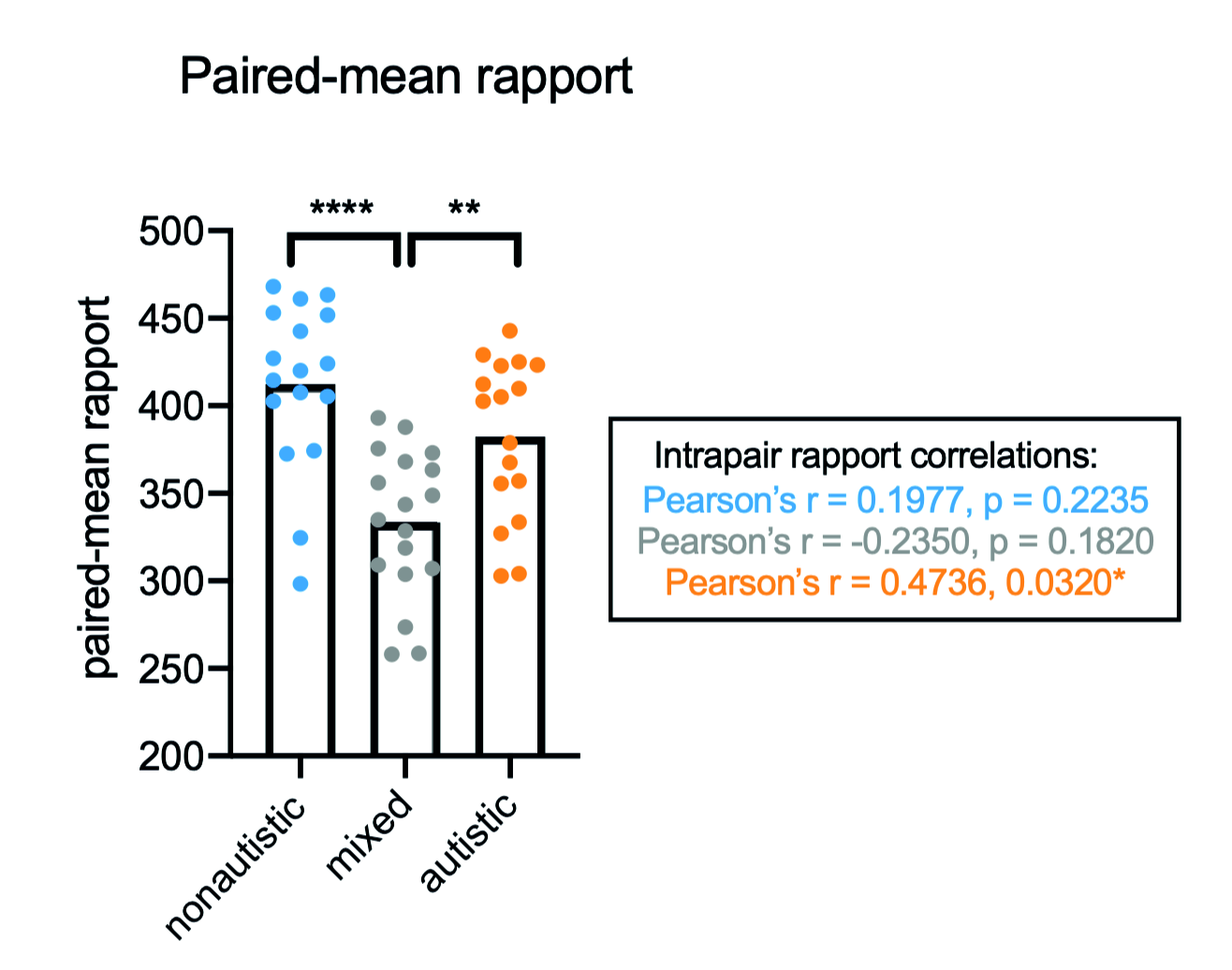


Supplementary Figure 1: Paired-mean rapport in non-autistic, mixed and autistic pairs. Intrapair rapport correlations for non-autistic (blue), mixed (grey) and autistic (orange) groups are shown on the right (* = p < 0.05, ** = p < 0.01, *** = p < 0.001, **** = p < 0.0001).
