# Supplementary material for: Investigating Markers of Rapport in Autistic and Nonautistic Interactions
Source: Autism Adulthood. 2022 Mar 9;4(1):3–11. doi: 10.1089/aut.2021.0017 (PMC8992924; doi:10.1089/aut.2021.0017)
Supplement: Supplemental data [file Supp_FigS2.docx]

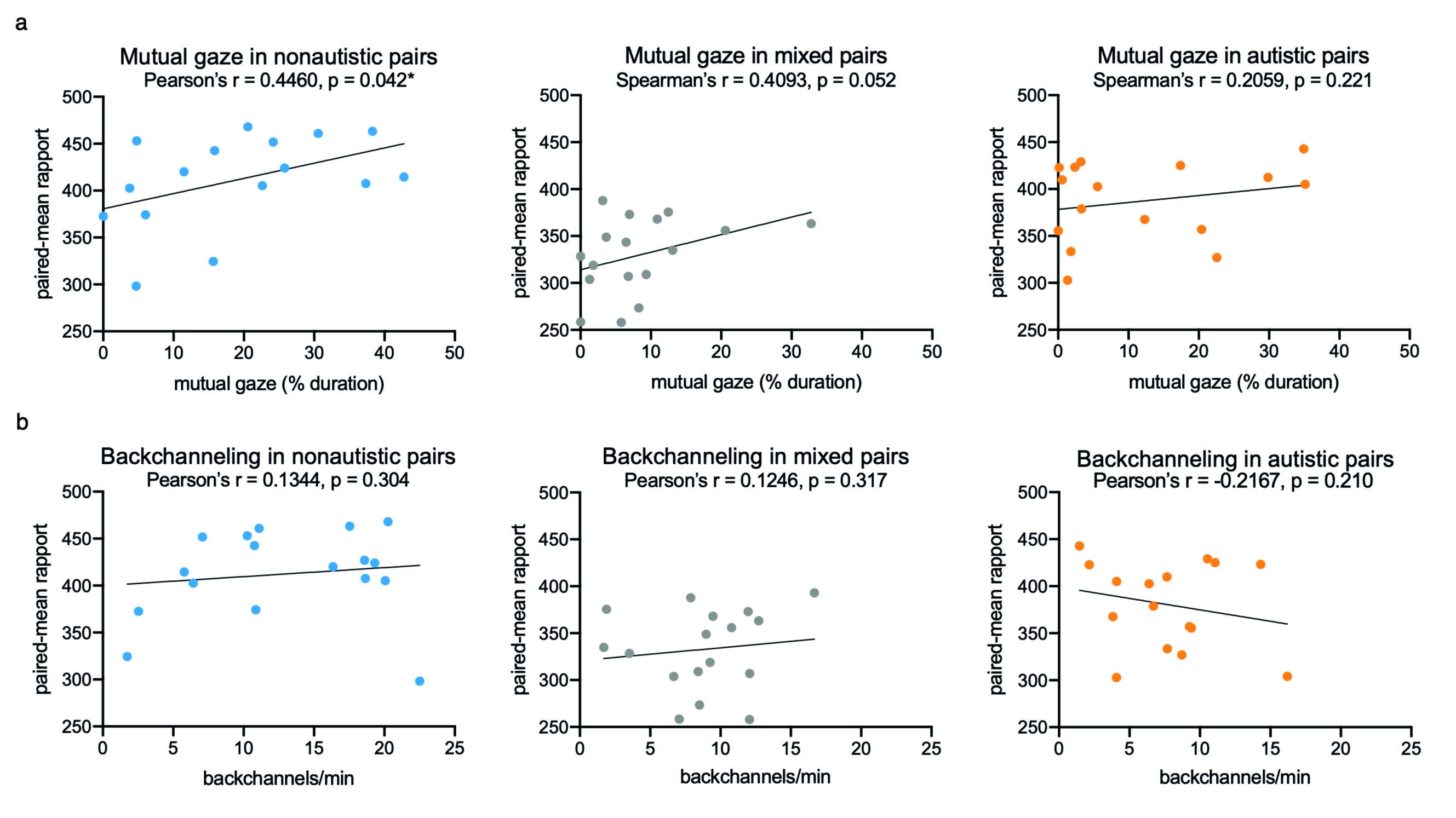


Supplementary Figure 2: Correlations between candidate rapport markers and paired-mean rapport in non-autistic, mixed and autistic pairs. (a-b): correlation coefficients and best-fit line shown by pair type for (a) mutual gaze duration % and (b) total backchannelling rate (backchannels/min).
